# Prevalence of Futility Protocols for Severely Bleeding Trauma Patients: A Survey from the Association for the Advancement of Blood & Biotherapies (AABB)

**DOI:** 10.3390/jcm15041541

**Published:** 2026-02-15

**Authors:** Samuel J. Thomas, Dan A. Waxman, Daniela Hermelin, Elizabeth Hartwell, Jed B. Gorlin, Sharon Carayiannis, Srijana Rajbhandary, Connor M. Bunch, Joseph B. Miller, Jeffrey L. Johnson, Ileana Lopez-Plaza, Rachel L. Brancamp, Ernest E. Moore, Hunter B. Moore, Peter K. Moore, Scott G. Thomas, Donald F. Zimmer, Mahmoud D. Al-Fadhl, Mark M. Walsh, Futile Indicators for Stopping Transfusion in Trauma (FISTT) Group

**Affiliations:** 1 Department of Emergency Medicine, Saint Joseph Regional Medical Center, Mishawaka, IN 46545, USA; samueljthomas1@gmail.com (S.J.T.); mahmoud8828@gmail.com (M.D.A.-F.); 2 Department of Transfusion Medicine Blood Services, Versiti Blood Center of Indiana, Indianapolis, IN 46208, USA; dwaxman@versiti.org; 3 Medical Affairs, ImpactLife Blood Center, Davenport, IA 52807, USA; dhermelin@impactlife.org; 4 Department of Pathology, Saint Louis University School of Medicine, St. Louis, MO 63104, USA; 5 Department of Pathology & Immunology, Baylor College of Medicine, Houston, TX 77030, USA; b.hartwell@sbcglobal.net; 6 Memorial Blood Centers, Minneapolis, MN 55402, USA; jed.gorlin@innovativeblood.org; 7 Association for the Advancement of Blood & Biotherapies, Bethesda, MD 20814, USA; scarayiannis@aabb.org; 8 Pubra Labs & Consulting, Fairfax, VA 22031, USA; srijana.rajbhandary1@gmail.com; 9 Division of Critical Care, Departments of Emergency Medicine and Internal Medicine, Henry Ford Hospital and Michigan State University Health Sciences, Detroit, MI 48202, USA; cbunch1@hfhs.org (C.M.B.);; 10 Division of Acute Care Surgery, Department of Surgery, Henry Ford Hospital and Michigan State University Health Sciences, Detroit, MI 48202, USA; jjohns52@hfhs.org; 11 Division of Blood Banking, Department of Pathology, Henry Ford Hospital and Michigan State University Health Sciences, Detroit, MI 48202, USA; iplaza@hfhs.org (I.L.-P.); rbranca2@hfhs.org (R.L.B.); 12 Ernest E. Moore Shock Trauma Center, Denver Health, Denver, CO 80204, USA; ernest.moore@dhha.org; 13 Department of Surgery, University of Colorado Anschutz School of Medicine, Aurora, CO 80045, USA; 14 AdventHealth Transplant Institute Porter, Denver, CO 80210, USA; hunterbmoore@gmail.com; 15 Department of Medicine, University of Colorado Anschutz School of Medicine, Aurora, CO 80045, USA; peter.moore@cuanschutz.edu; 16 Department of Surgery, Memorial Hospital, Beacon Medical Group Trauma & Surgical Services, South Bend, IN 46601, USA; sthomas@beaconhealthsystem.org (S.G.T.); dzimmer@beaconhealthsystem.org (D.F.Z.); 17 Department of Emergency Medicine, Indiana University School of Medicine, South Bend, IN 46617, USA

**Keywords:** blood banking, medical futility, futile resuscitation, massive transfusion, trauma, biomarkers, whole blood, clinical protocols, blood conservation strategies, health care surveys

## Abstract

**Background/Objectives**: The United States is facing a national blood shortage, which is a function of the reduced number of donors since the COVID-19 pandemic and the increasing use of balanced hemostatic resuscitation for severely bleeding trauma patients. As a result, recent attempts to define futility based on clinical and laboratory criteria have been proposed. There is no literature on the frequency of institutional futility protocols, either at hospitals or blood collection centers. **Methods**: The Association for the Advancement of Blood & Biotherapies sent out a survey to 800 United States hospitals and blood collection centers to determine the frequency of trauma futility protocols and the need to limit blood for non-trauma patients due to high use in trauma patients. **Results**: 213 (26.6%) institutions responded. 10.8% of hospitals and blood collection centers reported having a trauma futility protocol, and those hospitals and blood collection centers with futility protocols were more likely to have needed to limit blood to non-trauma patients due to high consumption by trauma patients. **Conclusions**: Trauma futility protocols at hospitals and blood collection centers are uncommon. Because of the national shortage of blood products available for trauma and non-trauma cases, implementing institutional trauma futility protocols may help to curb the incidence of blood limitation to non-trauma patients. Increased awareness and communication between blood bankers and traumatologists during the declaration of futility may reduce blood wastage and enhance the nation’s blood supply reservoirs.

## 1. Introduction

Following the COVID-19 pandemic in 2019, the already strained blood supply in the United States came under increasing pressure. This was driven in part by balanced hemostatic resuscitation using a 1:1:1 ratio of packed red blood cells (PRBCs), plasma, and platelets, as well as more frequent use of whole blood for patients with severe hemorrhage during trauma resuscitation [1,2,3,4,5,6,7,8]. Additionally, traumatologists may over-transfuse SBTPs due to the lack of clear thresholds for stopping transfusion, leading to unnecessary wastage and fewer blood products available for non-trauma patients [9,10]. These practices, while potentially lifesaving, substantially increase blood product utilization in a small subset of severely bleeding trauma patients (SBTPs), intensifying stress on limited inventories.

Periodic blood shortages during periods of peak trauma have been reported in the literature before and after the COVID-19 pandemic, and it has been shown that shortages of specific blood products caused by trauma resuscitation can limit product availability for simultaneous use of blood products for other SBTPs [9,11,12,13,14]. The shortages are driven by high-volume resuscitation and competing demands from elective and emergency surgery. Importantly, there has been little attention to the effect of trauma-related surges on blood availability for non-trauma cases [13]. In addition, it has been estimated that there are wastage rates up to 9% for PRBCs, 7% for plasma, 7% for platelets, and 33% for cryoprecipitate for severe trauma resuscitation at United States Level I trauma centers [9].

As a result, traumatologists and transfusion services have renewed efforts to seek definitive and highly predictive measurements of futility for SBTPs who are unlikely to survive beyond the initial hours of resuscitation, despite aggressive massive transfusion. Historically, establishing objective criteria for SBTPs has proven to be challenging; however, recent blood supply constraints have revived interest in identifying reliable, clinically actionable indicators that accurately determine futility in these cases [1,2,4,5,15,16,17,18,19,20,21,22,23,24,25].

At present, there are no nationally accepted protocols defining when massive transfusion should be discontinued for SBTPs who fail to respond to hemostatic resuscitation during the early hours of resuscitation. The latest American College of Surgeons Trauma Quality Improvement Program (ACS-TQIP) guidelines state that massive transfusion in trauma should be suspended when any further efforts are futile and lists transfusion cut-points and viscoelastic parameters that may signify futility [26]. However, these recommendations predate both the COVID-19 pandemic and the widespread adoption of modern balanced hemostatic resuscitation and whole blood strategies, which significantly affected blood supplies. Subsequent studies based on ACS-TQIP data continue to emphasize the importance of determining futility for SBTPs who do not respond to resuscitation but acknowledge that this goal has remained elusive [5,24]. Multiple investigators have proposed clinical, physiologic, and laboratory predictors, including Glasgow Coma Scale (GCS), severe hypotension, lactic acidosis, cardiorespiratory arrest with return of spontaneous circulation (ROSC), and severe fibrinolysis on viscoelastic testing, as reliable predictors of futility [2,27]. Trauma futility protocols to establish when to withdraw care for SBTPs have recently evolved to incorporate reliable clinical and laboratory markers used to guide resuscitation, with some controversy regarding the use of blood components thresholds as markers for futility [3,5,19,20,24,27,28].

Ongoing transfusion of blood products for SBTPs who are unlikely to survive may directly contribute to shortages that limit the availability of blood products for simultaneous or future non-trauma use. In response, the Association for the Advancement of Blood & Biotherapies (AABB) petitioned United States AABB-accredited blood collection centers and hospital blood banks in 2024 to assess existing and planned futile resuscitation practices in trauma. The goal of this study is to determine the frequency of blood product shortages caused by trauma resuscitation for AABB-accredited hospitals and blood centers and to assess the prevalence of institutional trauma futility protocols.

## 2. Materials and Methods

### 2.1. Study Design

A survey was designed by a group of AABB-associated blood collection centers directors and hospital transfusion medicine specialists. The target population was all blood collection centers and hospital blood banks that supply blood component therapy in the United States. The full survey is shown in Supplementary Materials.

An expert panel of traumatologists, trauma surgeons, blood bank directors, anesthesiologists, emergency physicians, pathologists, and clinical laboratory specialists collaborated with AABB quality assurance and survey personnel to design and refine a survey that aimed to collect data on the institutions’ futile resuscitation practices, such as the use of clinical or laboratory markers to determine whether suspension of the transfusion of blood products to a SBTP is advisable.

In addition to determining the type of institution (blood collection center, trauma center, university, federal government hospital, community hospital) and role of respondent (blood bank supervisor, medical director of blood bank, medical director of transfusion service), the survey sought to answer three main questions:

1. Does the institution have a resuscitation protocol with a consideration for futility to guide the suspension of blood product administration during massive trauma resuscitation?
2. For the institutions without a futility protocol, is the institution planning to develop or implement a futility protocol in the near future?
3. Over the past 12 months, has the institution ever limited the availability of blood products to patients outside of the trauma setting due to high use of blood transfusions in massive trauma resuscitation efforts?

Additional questions, which further delineated the specific parameters that the futility protocols included, were also included in the survey.

### 2.2. Survey Administration

The initial survey invitation was distributed via email to 2985 individuals at blood centers and hospitals. With the aim of receiving responses beyond AABB institutional membership, individual members associated with non-accredited AABB institutions were also sent initial survey invitations. However, because of uniform non-response from these individuals and difficulty in tracking their affiliations, these individuals were removed from the denominator. Additionally, contacts at affiliated hospitals that shared blood banks were filtered to prevent duplicate responses. Therefore, of the 1431 hospitals and blood centers initially traced, non-AABB-accredited institutions and duplicates were excluded, bringing the survey denominator to 800 AABB-accredited blood collection centers and hospital blood banks. The survey was distributed to contacts at AABB-member facilities, but the contacts were asked to forward the survey to the appropriate colleague if there was someone else who was better suited to answer the survey questions.

The AABB Futile Resuscitation Protocol Survey was developed and administered using the Qualtrics survey platform. The survey was open for responses from 16 September 2024, through 25 October 2024. The original closing date of 27 September 2024, was extended to increase participation.

A total of six reminder emails were sent to non-responders during the data collection period to encourage participation. In total, 225 completed survey responses were received, representing 213 unique institutions.

### 2.3. Data Collection and Study Preparation

The AABB executed a data sharing agreement and shared the data with all the co-authors to allow data analysis once the data were collected. The responses were identifiable in the database as self-reported institutions and positions provided by each respondent. The data were input into a Microsoft Excel (Version 16.101.3, Redmond, WA, USA) spreadsheet. Twelve duplicate responses from the same institutions were removed, reducing the number of respondents from 225 to 213, and all responses were deidentified for analysis.

### 2.4. Ethical Considerations

Institutional Review Board approval was requested, but since the survey did not constitute human subjects research, the approval was waived. A privacy review was performed to ensure that system data requirements were met prior to starting the study.

### 2.5. Analysis

Respondents were first analyzed as an aggregate and then split into three groups based on institution type. The first group consisted of Level I, II, III, and IV trauma centers, whether community, federal, or university, and blood banks located at those institutions. The second group was made of hospitals, whether community, federal, or university, that did not report as trauma centers and blood banks located at those institutions. The third group was composed of independent blood collection centers not based at hospitals. Blood collection center refers to the freestanding blood suppliers not belonging to trauma or non-trauma hospital centers, whereas hospital blood banks belong to trauma or non-trauma hospital centers. Furthermore, analysis of Level I, II, III, and IV trauma centers as separate groups was also performed. To safeguard the confidentiality of AABB-accredited member institutions, the data were not analyzed for geographic or demographic trends.

Analysis in Microsoft Excel was performed to determine the percentage of institutions that limited blood products for non-trauma patients during periods of peak trauma in the past 12 months, that exhibited futility protocols, and that planned to create a futility protocol. Additional analysis was done to determine what parameters those institutions with futility protocols used when determining futility for SBTPs.

## 3. Results

Of the 800 AABB-accredited blood centers, 213 (26.6%) responded to the survey concerning futile resuscitation for SBTPs. A summary of respondents is presented in Table 1.

Analysis of trauma centers, non-trauma centers, and freestanding blood collection centers was performed, along with aggregate analysis of all institutions surveyed. A summary of the survey results is presented in Table 2.

In addition to the key point that only 10.8% of all institutions had a trauma futility protocol, it was noted that of the institutions that did not have a futility protocol but planned to implement one, 23.5% had experienced the need to limit blood to non-trauma patients in the preceding year. Of the institutions that did not have a futility protocol and did not plan to implement one, only 6.9% had experienced the need to limit blood to non-trauma patients in the preceding year, suggesting that experience with recent blood shortage may be an important motivation for institutions to implement a futility protocol.

After comparing trauma centers to other types of institutions, trauma centers were split into subgroups based on level designation (I, II, III, or IV) to compare different levels against each other. Sub-analysis of the trauma centers is presented in Table 3.

The 23 surveyed institutions with a trauma futility protocol had different clinical, laboratory, and transfusion threshold measurements considered relevant for declaring futility for SBTPs. The most cited clinical measurements were absent signs of life, cardiac arrest, mechanism of injury, and shock or reverse shock index, while prothrombin time (PT)/international normalized ratio (INR), hemoglobin/hematocrit, partial thromboplastin time (PTT), lactic acid, and platelet count were the most common laboratory measurements. A full summary of futility measurements is presented in Table 4.

Table 4 reflects the heterogeneity of clinical and laboratory measurements used to define futility among those institutions that have futility protocols. There was a prevalence of specific clinical and laboratory markers used to predict futility, but no marker was universal. The most common measurement was PT/INR with a 60.9% prevalence.

## 4. Discussion

### 4.1. Observations and Applications of the Survey

This survey demonstrates that, despite the pressures of the COVID-19 pandemic reducing the national blood supply and the increased use of balanced hemostatic resuscitation with or without whole blood, trauma futility protocols remain uncommon across United States blood banks. Among 213 AABB-accredited institutions, only 10.8% had a trauma futility protocol, and only 8.9% of institutions without a protocol planned to develop or implement a protocol in the future. However, only 10.3% of institutions reported ever needing to limit blood to non-trauma patients because of high trauma utilization in the preceding year. This likely contributes to institutional inertia, as centers that have not experienced shortage may not perceive futility protocols to be necessary [29]. In contrast, institutions that have faced problems with blood shortage before or those that are likely to face blood shortage in the future might feel the need to have a protocol in place, suggesting that real-world scarcity drives policy adoption. This may be a reason why 26.1% of institutions with a futility protocol reported needing to limit blood to non-trauma patients due to trauma resuscitation, compared to 8.4% of institutions without futility protocols. This assumption is supported by the fact that 23.5% of institutions that planned to implement a futility protocol in the future had experienced a shortage of blood products for non-trauma patients in the past 12 months, while only 6.9% of institutions that did not plan to implement a protocol had to limit blood in the past 12 months. This suggests that institutions are more likely to plan to implement a futility protocol for trauma if they had experienced the need to limit blood products to other patients before and that these protocols are being implemented to curb transfusion in the trauma setting.

Compared to non-trauma hospitals and freestanding blood banks, more trauma centers reported having a trauma futility protocol or planning to implement one, and more trauma centers reported needing to limit blood to non-trauma patients due to high usage during trauma resuscitation. Similarly, more Level I and II trauma centers, which experience a higher volume of trauma cases, reported having a trauma futility protocol in place than Level III and IV trauma centers. This suggests that trauma futility protocols may be more common at trauma centers and at those trauma centers that observe higher rates of trauma cases (Level I and II centers) because they might be more likely to observe blood shortage during peak trauma. Therefore, trauma futility protocols may be seen as more necessary at the trauma centers that experience a higher volume of trauma cases and are more likely to run out of blood. However, of the 16 total trauma centers that had a futility protocol, 12 (75%) did not report needing to limit blood to other non-trauma patients in the past 12 months. This could suggest that implementing a trauma futility protocol could be associated with a decreased need to limit blood products to other patients, assuming that trauma institutions with futility protocols are more likely to face the possibility of a blood shortage. This may reflect on the futility protocols’ success in limiting blood product wastage, which has been a chronic problem for trauma centers [9,30]. However, this survey alone cannot demonstrate causation between the implementation of trauma futility protocols and reduced blood usage in the trauma setting, although recent literature has documented the need for futility protocols to limit blood wastage [2,4,17,19,20].

Many trauma centers in the survey did not have a futility protocol, and this may indicate that at these trauma centers, the necessity for limiting blood products is not as frequent as in those trauma centers that have a futility protocol. And even though non-trauma hospitals and freestanding blood collection centers did not frequently report needing to limit blood, having a futility protocol, or planning to develop one in the future, there was at least one institution of each type that did. This reflects the widespread effects of massive trauma transfusion, as even institutions that do not regularly treat trauma patients have their blood supplies affected by trauma due to the need to transport and allocate blood to trauma hospitals [23,31].

Analysis of the different parameters that institutions use in futility protocols reveals that there is high use of futility time-outs (34.8%) to pause resuscitation and make a team-based decision to consider stopping blood transfusion when efforts seem futile. This reflects recent trauma literature, which has called for futility time-outs to be implemented in trauma protocols [2,20,23]. Specific clinical parameters that are highly reported include cardiac arrest (47.8%), mechanism of injury (43.5%), shock index/reverse shock index (43.5%), systolic blood pressure (34.8%), and severity of head injury/GCS (34.8%), which are all highly cited in the trauma literature as significant predictors of mortality [2,27,32,33,34]. Notably, age was not often reported as a trauma futility parameter by the respondents of this survey (13.0%), although it is a commonly cited predictor of futility [27,33,34,35,36]. Previous literature has shown that children are less likely to undergo withdrawal of resuscitation efforts, since they are more likely to survive, even with high transfusion volumes and presence of traumatic brain injury [22,37,38]. Highly reported laboratory parameters include PT/INR (60.9%), hemoglobin/hematocrit (52.2%), PTT (52.2%), lactic acid (47.8%), and platelet count (47.8%), which reflect commonly cited parameters in the trauma literature [2,34,39]. The amount of PRBCs transfused was the most reported blood transfusion threshold (30.4%), yet overall, blood transfusion thresholds were not commonly reported as being used in trauma futility protocols. This reflects the controversy in the literature regarding the use of transfusion cut-points to determine futility [3,5,19,20,24,27,28]. Importantly, no single parameter was universally employed by all institutions that reported having a current futility protocol.

The most salient point of this survey is that futility protocols, whether at trauma hospitals, non-trauma hospitals, or blood collection centers, are still fairly uncommon. Futility protocols are still mostly limited to trauma centers and remain relatively infrequent despite the recent urgency created by national blood shortages and the recent increased interest in developing reliable futility markers for SBTPs.

### 4.2. Limitations and Future Directions

One limitation of this survey is that the response rate of 26.6% may skew the estimated prevalence of institutions that have adopted futility protocols. Filling out the survey took time and labor for the respondents and relied on the AABB contact to respond to the initial invitation and one of the subsequent six reminders to fill out the survey. The blood banking and health care industries are facing greater workforce reductions and more demands on their time [40,41,42]. That may be why, despite six reminder emails sent to non-responders, approximately 63% of sampled hospitals and blood centers did not respond to the survey. A second reason for non-response may be that the concept of defining futility for SBTPs who are unlikely to survive resuscitation has only recently become prominent in the transfusion literature, and as the results of the survey have demonstrated, few institutions and trauma centers have futility protocols in place. Future, more targeted surveys may reveal an increased response rate as more institutions become familiar with the concept of adopting a futility protocol for SBTPs unlikely to respond to resuscitation. Another limitation is that the survey was sent out early in the implementation of whole blood in hospital and pre-hospital resuscitation and may not accurately reflect the future that whole blood resuscitation will have on blood shortages.

Additionally, this survey did not consider the impact of organ procurement organization (OPO) consultation on futility protocols so as to not dilute the main goal of the survey, which was to determine the frequency of trauma futility protocols and blood shortages for non-trauma patients due to trauma. Future surveys may include the frequency of OPO consultation for those SBTPs who face futility in the first 4 h and the mechanism of injury (penetrating or blunt) and age of the potential donor, as there is a clear lack of consensus regarding the timing of the early consultation of consulting OPO when resuscitation is futile [26,33,34,37,43,44,45,46,47,48,49,50,51]. This will be particularly important for uncontrolled donation after circulatory death, whereby early notification and arrival of the OPO staff will face the barrier of the short amount of time available to evaluate the eligibility and carry out the necessary diagnostic and therapeutic interventions to keep the patient alive long enough for safe organ procurement [37,52,53,54,55,56].

Likewise, future studies could look at the Patient Blood Management (PBM) performance improvement process. The goal of PBM is to limit the amount of blood products transfused by improving red blood cell mass, limiting bleeding, and using a transfusion threshold for carefully selected patients. These goals have historically been applied most often in the perioperative period [57]. However, in addition to the perioperative period, another area for the application of PBM is the conservation of blood products by using futility protocols in the early moments of trauma resuscitation for SBTPs [58]. Future surveys could investigate the prevalence of PBM practices and PBM committees to help implement and monitor futility protocols for SBTPs.

### 4.3. Proposal for Expanded Implementation of Trauma Futility Protocols

The effect of the increased use of blood products during trauma on other trauma patients at the same time has been demonstrated [13]. This survey expands that observation to include the effect of trauma-induced blood shortages on the non-trauma patient population as well. Because of the fungible nature of blood products whereby freestanding blood centers and non-trauma hospitals often provide blood products to trauma centers during times of need, this survey provides evidence that the previously mentioned shortages caused by trauma affect not only other trauma patients but also non-trauma patients. It has been noted that there have been multiple strategies to practice hypervigilance in the distribution of blood products during times of peak trauma, which include transferring inventory internally among the same enterprise or transferring to other institutions, as well as prioritizing allocation to certain patient populations who may benefit from continued aggressive resuscitation and might not face a transfusion cut-point to define futility, such as young patients with penetrating injury [5,23,24,31].

Because blood banks control the distribution of blood products across all clinical services, awareness of futility protocols by the blood bank allows the blood bank to better communicate competing demands for a limited inventory. When high volumes of blood are being transfused to a SBTP who has failed to respond to initial resuscitation and who prognosis is poor, those products are no longer available for other patients in the same hospital who may have a substantially higher likelihood of benefits.

The blood bank director and staff would be better able to effectively distribute blood products within the hospital if they were part of the decision-making process for withdrawing care and by referring to the futility protocol to discuss the need to withdraw care with the trauma surgeon, anesthesiologist, intensivist, or emergency physician. This same philosophy may also be applied to non-trauma centers and blood collection centers, since blood products can be allocated from these centers to trauma centers [23,31]. This strategy is based on the principle that blood management functions not as an isolated repository of blood products within an institution but as an integrated system within an institution and among partnering hospitals and blood centers. The low number of independent blood collection centers that have a designated trauma futility protocol indicates that many blood collection centers might not consider futility when allocating blood products to different institutions during peak trauma. Therefore, blood collection centers may benefit from communication with trauma centers about both the futility of the trauma patient and the need for blood elsewhere in the system.

Transfusion service strategies should allow for the maximized use of the limited blood inventory available during periods of peak trauma. These efforts could be communicated with important stakeholders with continued education and refining of the standards of futility within the context of institutional quality control [5,10,24,31,49]. Therefore, increased awareness and education concerning the nationwide blood shortage during periods of peak trauma should induce all blood banking centers, including trauma centers, non-trauma center hospitals, and blood collection centers, to implement protocols for declaring futile resuscitation for SBTPs. The presence of a futility protocol would provide a more definitive foundation upon which the traumatologists and blood bankers could ascertain when to stop transfusion for trauma patients. ACS-TQIP guidelines state that the decision to stop transfusion for SBTPs should be a shared decision between the trauma surgeon and either the anesthesiologist if in the operating room or the intensivist if in the intensive care unit [26]. However, the blood banker should also be consulted in real time when futility measures are undertaken to provide important insights regarding the reservoir of blood products available not just for a particular trauma but also for other trauma and non-trauma cases [23,31]. It has also been proposed that designated transfusion teams direct the utilization of blood components for SBTPs [30]. In addition to these major stakeholders, a PBM committee and an ethics committee may find it important to participate not only in the formulation of futility guidelines for SBTPs but also in periodic review of the protocol. A preemptive collaboration between the blood bank, trauma surgery, anesthesia, and emergency medicine could be a useful collaboration to prevent further blood shortages for trauma and non-trauma patients [45].

Equally important as the medical decisions regarding futility are the ethical principles, which form the foundation upon which these difficult, time-pressured decisions regarding the withdrawal of care for SBTPs must be made [37,59]. The search for certainty regarding the decision to withdraw care for SBTPs by clinicians and ethicists has been supported by the use of markers or a combination of markers that have 100% positive predictive value and specificity for determining futility [2]. This certainty is driven not only by a desire for medical accuracy but also for the ethical standard that there should be no trauma patient who is deprived of resuscitation based on futility guidelines who would survive with continued resuscitation. The prognostic accuracy needs to be as close to 100% as possible. The equal pursuits of ethical and medical certainty require continued collaboration not just between medical specialties but also non-medical specialties, such as an ethics committee [45].

## 5. Conclusions

This survey demonstrates that trauma futility protocols at trauma hospitals, non-trauma hospitals, and blood collection centers are relatively uncommon, despite the well-recognized nationwide shortage of blood products during peak periods of trauma. The limitation of blood products to non-trauma patients due to high transfusion rates for trauma patients was uncommon but occurred in all three types of institutions. The implementation of institutional futility protocols in hospital blood banks and blood collection centers for trauma patients may help to limit the incidence of blood limitation to both trauma and non-trauma patients by establishing reliable guidelines for defining futility for SBTPs who have not responded to aggressive hemostatic resuscitation and whose prognosis is bleak.

## Figures and Tables

**Table 1:** This table shows a summary of the respondents of the survey, including the institution type and role of the person who filled out the survey. Percentages exceed 100% because respondents could select multiple categories.

Institution Type (May Be Concurrent)	Number of Institutions (N = 213) n (%)
Blood Bank/Center	133 (62.4)
Trauma Center	110 (51.6)
Level I	60 (28.2)
Level II	30 (14.1)
Level III	18 (8.5)
Level IV	6 (2.8)
University	30 (14.1)
Federal Government Hospital	45 (21.1)
Community Hospital	37 (17.4)
Other	4 (1.9)
**Institutional Role of Respondent (may be concurrent)**	**n (%)**
Blood Bank Supervisor	94 (44.1)
Medical Director of Blood Bank	31 (14.6)
Medical Director of Transfusion Service	47 (22.1)
Other	56 (26.3)

**Table 2:** This table shows the results of the survey for trauma center hospitals, non-trauma center hospitals, and freestanding blood collection centers.

Criteria	All Institutions (N = 213) n (%)	Trauma Center Hospitals and Blood Banks (N = 110) n (%)	Non-Trauma Center Hospitals and Blood Banks (N = 66) n (%)	Non-Hospital Blood Collection Centers (N = 37) n (%)
**Current Trauma Futility Protocol**	**23 (10.8)**	**16 (14.5)**	**6 (9.1)**	**1 (2.7)**
Recent Need to Limit Blood to Non-Trauma Patients in the Past Year	6 (26.1)	4 (25.0)	2 (33.3)	0 (0)
**No Current Trauma Futility Protocol**	**190 (89.2)**	**94 (85.5)**	**60 (90.9)**	**36 (97.3)**
Recent Need to Limit Blood to Non-Trauma Patients in the Past Year	16 (8.4)	12 (12.8)	3 (5.0)	1 (2.8)
Planned Trauma Futility Protocol *	**17 (8.9)**	**15 (16.0)**	**1 (1.7)**	**1 (2.8)**
Recent Need to Limit Blood to Non-Trauma Patients in the Past Year	4 (23.5)	4 (26.7)	0 (0)	0 (0)
No Planned Trauma Futility Protocol *	**173 (91.1)**	**79 (84.0)**	**59 (98.3)**	**35 (97.2)**
Recent Need to Limit Blood to Non-Trauma Patients in the Past Year	12 (6.9)	8 (10.1)	3 (5.9)	1 (2.9)

* Percentages are calculated by dividing by the number of institutions without current trauma futility protocols.

**Table 3:** This table shows the results of the survey for trauma center hospitals, grouped by level.

Criteria	Level I (N = 60) n (%)	Level II (N = 30) n (%)	Level III (N = 18) n (%)	Level IV (N = 6) n (%)
**Current Trauma Futility Protocol**	**12 (20.0)**	**4 (13.3)**	**1 (5.6)**	**1 (16.7)**
Recent Need to Limit Blood to Non-Trauma Patients in the Past Year	2 (16.7)	1 (25.0)	1 (100)	0 (0)
**No Trauma Futility Protocol**	**48 (80.0)**	**26 (86.7)**	**17 (94.4)**	**5 (83.3)**
Recent Need to Limit Blood to Non-Trauma Patients in the Past Year	5 (10.4)	5 (19.2)	1 (5.9)	2 (40.0)
Planned Trauma Futility Protocol *	**10 (20.8)**	**1 (3.8)**	**4 (23.5)**	**1 (20.0)**
Recent Need to Limit Blood to Non-Trauma Patients in the Past Year	3 (30.0)	0 (0)	1 (25.0)	1 (100)
No Planned Trauma Futility Protocol *	**38 (79.2)**	**25 (96.2)**	**13 (76.5)**	**4 (80.0)**
Recent Need to Limit Blood to Non-Trauma Patients in the Past Year	2 (5.3)	5 (20.0)	0 (0)	1 (25.0)

* Percentages are calculated by dividing by the number of trauma centers of the same level designation without current trauma futility protocols.

**Table 4:** This table describes the clinical, laboratory, and blood product threshold measurements that are part of the futility protocols for SBTPs from the institutions that reported currently having a futility protocol in place.

Parameter Used in Institutional Trauma Futility Protocol	All Institutions with a Current Trauma Futility Protocol (N = 23) n (%)
Futility Time-outs	8 (34.8)
**Clinical Parameters**	**n (%)**
Mechanism of Injury	10 (43.5)
Absent Signs of Life	11 (47.8)
Heart Rate	9 (39.1)
Systolic Blood Pressure	8 (34.8)
Respiratory Rate	6 (26.1)
Temperature	5 (21.7)
End-tidal Carbon Dioxide	4 (17.4)
Oxygen Saturation	7 (30.4)
Severity of Head Injury/Glasgow Coma Scale	8 (34.8)
Shock Index and/or Reverse Shock Index	10 (43.5)
Age	3 (13.0)
Endotracheal Intubation	2 (8.7)
Inotrope Administration	7 (30.4)
Factor VII Administration	2 (8.7)
Resuscitative Endovascular Balloon Occlusion of the Aorta (REBOA)	3 (13.0)
Thoracotomy	2 (8.7)
Cardiac Arrest	11 (47.8)
Return of Spontaneous Circulation (ROSC)	8 (34.8)
Presence of Cirrhosis	1 (4.3)
**Laboratory Measurements**	**n (%)**
pH	9 (39.1)
Base Deficit/Base Excess	8 (34.8)
Lactic Acid	11 (47.8)
Hemoglobin/Hematocrit	12 (52.2)
Prothrombin Time (PT)/International Normalized Ratio (INR)	14 (60.9)
Partial Thromboplastin Time (PTT)	12 (52.2)
Platelet Count	11 (47.8)
Fibrinogen	10 (43.5)
Bicarbonate (HCO3−)	7 (30.4)
Serum Calcium	3 (13.0)
Serum Potassium	3 (13.0)
Viscoelastic Testing	9 (39.1)
**Blood Product Transfusion Thresholds**	**n (%)**
Whole Blood	1 (4.3)
Packed Red Blood Cells (PRBCs)	7 (30.4)
Platelets	4 (17.4)
Fresh Frozen Plasma (FFP)	4 (17.4)
Cryoprecipitate	2 (8.7)
Liquid Plasma	0 (0)
Prothrombin Complex Concentrate	1 (4.3)
Fibrinogen Concentrate	0 (0)

## Data Availability

The datasets presented in this article are not made readily available to protect the confidentiality of AABB-accredited member institutions. Requests to access the datasets should be directed to the corresponding author.

## Supplementary Materials

The following supporting information can be downloaded at: https://www.mdpi.com/article/10.3390/jcm15041541/s1.

## Abbreviations

The following abbreviations are used in this manuscript:

AABB	Association for the Advancement of Blood & Biotherapies
ACS-TQIP	American College of Surgeons Trauma Quality Improvement Program
FFP	Fresh frozen plasma
GCS	Glasgow Coma Scale
INR	International normalized ratio
OPO	Organ procurement organization
PBM	Patient Blood Management
PRBC	Packed red blood cell
PT	Prothrombin time
PTT	Partial thromboplastin time
REBOA	Resuscitative endovascular balloon occlusion of the aorta
ROSC	Return of spontaneous circulation
SBTP	Severely bleeding trauma patient
