# Supplementary material for: Prevalence of Futility Protocols for Severely Bleeding Trauma Patients: A Survey from the Association for the Advancement of Blood & Biotherapies (AABB)
Source: J Clin Med. 2026 Feb 15;15(4):1541. doi: 10.3390/jcm15041541 (PMC12941769; doi:10.3390/jcm15041541)
Supplement: Supplementary file 1 [file jcm-15-01541-s001.zip › jcm-4127552-supplementary.pdf]

Association for the Advancement of Blood & Biotherapies

Futile Resuscitation Protocol Survey

Demographic Section

Futile Resuscitation Protocol Survey

Recent literature among trauma specialists has proposed specific parameters to further refine the definition of "futility" for patients receiving large volumes of blood components during resuscitation for severe hemorrhagic shock who have unsurvivable injuries. In the trauma literature, the term "futility" has become an important concept given the recently documented blood product shortages following the COVID-19 pandemic. In addition, the increased adoption of 1:1:1 (RBCs:Platelets:FFP) blood component therapy as well as whole blood therapy for trauma and other causes of severe hemorrhage have put further strain on blood banks to furnish blood products.

This survey aims to collect data on your institution's futile resuscitation practices, such as using clinical or laboratory markers to determine whether suspension of the

transfusion of blood products to a severely bleeding trauma patient is advisable.

Thank you for taking the time to fill out this survey. We appreciate your input and effort.

Our Research Question:
Is there a standard practice within trauma services or hospital-based centers with severely bleeding patients to assess and manage futility in the adult massive transfusion setting?

Target Population:
All hospitals and blood banks which supply blood component therapy

* Indicates required question

*1. With which institution/trauma center are you affiliated?

*2. Please characterize your institution/healthcare system. Select all that apply.

- ☐ Blood Bank
- ☐ Level I Trauma Center
- ☐ Level II Trauma Center
- ☐ Level III Trauma Center
- ☐ Level IV Trauma Center
- ☐ Level V Trauma Center
- ☐ University
- ☐ Community (Nonfederal acute care)
- ☐ Federal Government Hospital
- ☐ [ ] Other

*3. Which best describes your current position at your institution? If the Medical Director is not responsible, please refer the survey to the responsible party. At some institutions, trauma coordinators, trauma directors and/or nurse coordinators may be the most knowledgeable about the survey particulars. Select all that apply.

- ☐ Medical Director of Transfusion Service
- ☐ Medical Director of Blood Bank
- ☐ Blood Bank Supervisor

☐ [ ] Other

*4. Over the past 12 months, has your institution limited the availability of blood products to patients outside of the trauma setting due to high use of blood transfusions in massive trauma resuscitation efforts?

○ Yes
○ No
○ Not Sure

*5. Does your institution have a resuscitation protocol with a consideration for futility to guide suspension of blood product administration during massive trauma resuscitation?

○ Yes Skip to question 7
○ No Skip to question 6
○ Not Sure Skip to question 7

Possibility of Futile Resuscitation Protocol

*6. Does your institution plan to develop or implement a futility protocol in the near future?

- ○ Yes   Display question 15
- ○ No
- ○ Not Sure

Futile Resuscitation Protocol Criteria

In the following questions, please describe your institution's current practices in treating severely bleeding patients.

7. Futility ”time outs” are instances where the care team will pause protocol to assess likelihood of survival based on clinical and laboratory parameters including number of blood products transfused per hour.

Are futility “time outs” designated as part of your institution’s protocol?

- ○ Yes
- ○ No
- ○ Not Sure
- ○ Not Applicable

8. Does your institution utilize a futility index, physician gestalt, or scoring system in determining futility in ongoing resuscitation efforts for patients requiring massive transfusion?

- ○ Yes
- ○ No
- ○ Not Sure
- ○ Not Applicable

9. As per your institution's protocol, which of the following clinical parameters are used to determine futility in massive transfusion? Select all that apply.

- ☐ Mechanism of injury
- ☐ Absent signs of life
- ☐ Heart rate
- ☐ Systolic blood pressure
- ☐ Respiratory rate
- ☐ Temperature
- ☐ End-tidal carbon dioxide
- ☐ Oxygen saturation
- ☐ Severity of head injury
- ☐ Shock Index and/or Reverse Shock Index

- ☐ Age
- ☐ Endotracheal intubation
- ☐ Inotrope administration
- ☐ Factor VII administration
- ☐ Resuscitative Endovascular Balloon Occlusion of the Aorta (REBOA)
- ☐ Thoracotomy
- ☐ Cardiac arrest
- ☐ Return of Spontaneous Circulation (ROSC)
- ☐ None/Not Applicable
- ☐ ________ Other

10. As per your institution's protocol, which laboratory parameters are used to determine futility in massive transfusion? Select all that apply.

- ☐ pH
- ☐ Base deficit/base excess
- ☐ Lactic acid
- ☐ Hemoglobin/hematocrit
- ☐ Prothrombin Time/International Normalized Ratio (INR)
- ☐ Partial thromboplastin time (PTT)
- ☐ Platelet count
- ☐ Fibrinogen
- ☐ Bicarbonate (HCO3-)
- ☐ Serum Calcium

- [ ] Serum Potassium
- [ ] None/Not Applicable
- [ ] ______ Other

11. Does your institution utilize a viscoelastic testing algorithm (e.g. Quantra,TEG, ROTEM) to help determine futility in massive transfusion settings?

- ( ) Yes
- ( ) No
- ( ) Not Sure
- ( ) Not Applicable

12. For which of the following blood components do you have a cut-off point to determine suspension of resuscitation efforts? Select all that apply.

- [ ] Whole blood
- [ ] Packed red blood cells (pRBCs)
- [ ] Platelets
- [ ] Fresh frozen plasma (FFP)
- [ ] Cryoprecipitate
- [ ] Liquid plasma
- [ ] Prothrombin complex concentrate

- [ ] Fibrinogen concentrate
- [ ] We do not utilize a blood component cut-off point for our futility protocol
- [ ] Not Applicable

*13. Does your institution use the number of red blood cells transfused per hour as a transfusion cutpoint in assisting to determine futile resuscitation?

- ○ Yes — If this option is selected, please describe the cutpoint in the next question
- ○ No
- ○ Not Applicable

14. If your institution uses the number of red blood cells transfused per hour as a transfusion cutpoint in assisting to determine futile resuscitation, please describe the cutpoint. (Please include your institution's definition of a unit of blood)

Potential Futile Resuscitation Protocol Criteria

Please answer these questions to the best of your ability as we know that these decisions are not final.

15. Futility ”time outs” are instances where the care team will pause protocol to assess likelihood of survival based on clinical and laboratory parameters including number of blood products transfused per hour.

Would your institution consider futility “time outs” as part of your institution’s protocol?

- ○ Yes
- ○ No
- ○ Not Sure
- ○ Not Applicable

16. Would your institution consider utilizing a futility index, physician gestalt, or scoring system in determining futility in ongoing resuscitation efforts with patients requiring massive transfusion?

- ○ Yes
- ○ No
- ○ Not Sure
- ○ Not Applicable

17. Would your institution consider using the following clinical parameters to determine futility in massive transfusion? Select all that apply.

- ☐ Mechanism of injury
- ☐ Absent signs of life
- ☐ Heart rate
- ☐ Systolic blood pressure
- ☐ Respiratory rate
- ☐ Temperature
- ☐ End-tidal carbon dioxide
- ☐ Oxygen saturation
- ☐ Severity of head injury
- ☐ Shock Index and/or Reverse Shock Index
- ☐ Age
- ☐ Endotracheal intubation
- ☐ Inotrope administration
- ☐ Factor VII administration
- ☐ Resuscitative Endovascular Balloon Occlusion of the Aorta (REBOA)
- ☐ Thoracotomy
- ☐ Cardiac arrest

- [ ] Return of Spontaneous Circulation (ROSC)
- [ ] None/Not Applicable
- [ ] Other

18. Would your institution consider using the following laboratory parameters to determine futility in massive transfusion? Select all that apply.

- [ ] pH
- [ ] Base deficit/base excess
- [ ] Lactic acid
- [ ] Hemoglobin/hematocrit
- [ ] Prothrombin Time/International Normalized Ratio (INR)
- [ ] Partial thromboplastin time (PTT)
- [ ] Platelet count
- [ ] Fibrinogen
- [ ] Viscoelastic testing parameters (e.g. TEG LY30, ROTEM CL30, ROTEM CL60, and ML)
- [ ] Bicarbonate (HCO3-)
- [ ] Serum Calcium
- [ ] Serum Potassium
- [ ] None/Not Applicable
- [ ] Other

19. Would your institution consider utilizing a viscoelastic testing algorithm (e.g. TEG LY30,ROTEM CL30, ROTEM CL60, and ML) to help determine futility in massive transfusion settings?

- ○ Yes
- ○ No
- ○ Not Sure
- ○ Not Applicable

20. For which of the following blood components would your institution consider having a cutoff point to determine suspension of resuscitation efforts? Select all that apply.

- ☐ Whole blood
- ☐ Packed red blood cells (pRBCs)
- ☐ Platelets
- ☐ Fresh frozen plasma (FFP)
- ☐ Cryoprecipitate
- ☐ Liquid plasma
- ☐ Prothrombin complex concentrate
- ☐ Fibrinogen concentrate
- ☐ We will not utilize a blood component cut-off point for our futility protocol

21. Would your institution consider using the number of red blood cells transfused per hour as a transfusion cutpoint in assisting to determine futile resuscitation? Please describe the cutpoint. (Please include your institution's definition of a unit of blood, if applicable)
